# Sphingomyelin as a myelin biomarker in CSF of acquired demyelinating neuropathies

**DOI:** 10.1038/s41598-017-08314-1

**Published:** 2017-08-10

**Authors:** Giovanna Capodivento, Davide Visigalli, Martina Garnero, Roberto Fancellu, Michela Demetra Ferrara, Abdul Basit, Zeeshan Hamid, Vito Paolo Pastore, Silvano Garibaldi, Andrea Armirotti, Gianluigi Mancardi, Carlo Serrati, Elisabetta Capello, Angelo Schenone, Lucilla Nobbio

**Affiliations:** 1 Department of Neurosciences, Rehabilitation Ophthalmology, Genetics and Maternal-Infantile Sciences (DINOGMI) and CEBR, University of Genoa, Genoa, Italy; 2 Unit of Neurology, IRCCS San Martino University Hospital IST, Genoa, Italy; 3 Department of Drug Discovery and Development, Fondazione Istituto Italiano di Tecnologia, Genoa, Italy; 4 Neuroengineering and Bionanotechnology Lab (NBT), Department of Informatics, Bioengineering, Robotics and System Engineering (DIBRIS), University of Genoa, Genoa, Italy; 5 Division of Cardiology, IRCCS University Hospital San Martino, Research Centre of Cardiovascular Biology, University of Genoa, Genoa, Italy

## Abstract

Fast, accurate and reliable methods to quantify the amount of myelin still lack, both in humans and experimental models. The overall objective of the present study was to demonstrate that sphingomyelin (SM) in the cerebrospinal fluid (CSF) of patients affected by demyelinating neuropathies is a myelin biomarker. We found that SM levels mirror both peripheral myelination during development and small myelin rearrangements in experimental models. As in acquired demyelinating peripheral neuropathies myelin breakdown occurs, SM amount in the CSF of these patients might detect the myelin loss. Indeed, quantification of SM in 262 neurological patients showed a significant increase in patients with peripheral demyelination (p = 3.81 * 10 − 8) compared to subjects affected by non-demyelinating disorders. Interestingly, SM alone was able to distinguish demyelinating from axonal neuropathies and differs from the principal CSF indexes, confirming the novelty of this potential CSF index. In conclusion, SM is a specific and sensitive biomarker to monitor myelin pathology in the CSF of peripheral neuropathies. Most importantly, SM assay is simple, fast, inexpensive, and promising to be used in clinical practice and drug development.

## Introduction

Myelin disorders negatively influence wellness of patients for most of their life. Existing treatments may delay the disease progression and symptoms, but remyelination promoting compounds have been recently identified only in experimental studies^1^. Moreover, experience from completed and ongoing clinical trials and natural history studies confirms difficulties in detecting clinical changes for most of these disorders, making urgent the identification of sensitive-to-change outcome measures. In this context, biological markers can serve many unique purposes, including confirmation of diagnosis, monitoring disease progression or treatment effects, and prediction of clinical outcome. Biomarkers also play an important role in the preclinical assessment of potentially beneficial and harmful effects of new candidate drugs. Screening tests in animals using biomarkers provide important demonstration that a compound is likely to have the intended therapeutic activity in patients. The development of effective biomarkers, allowing a better prediction of the disease severity, will ensure a more precise understanding of the pathomechanisms, avoiding costly and lifelong unnecessary treatments^2^.

At present, the amount of myelin may be assessed only in the central nervous system (CNS) of human patients by sophisticated techniques^3, 4^. These systems are quite expensive and require extensive time commitment and specific expertise which make them unsuitable for daily clinical practice. Moreover, to the best of our knowledge, similar techniques are not employed in peripheral nervous system (PNS) diseases to rate the degree of myelination^5–7^. Furthermore, to realistically monitor myelin membranes in human patients, the only option is the identification of an easily detectable biomarker in biological fluids^8^. The higher lipid-to-protein ratio and unique lipid composition of myelin prompted us to focus on the lipid component of the myelin sheath^9^.

Here, we test sphingomyelin (SM), a myelin enriched lipid, by a fluorescence-based assay in different experimental settings to provide proof of concept that its levels are sensitive, specific and reliable to monitor peripheral myelination and myelin remodeling. Cerebrospinal fluid (CSF) of patients affected by immune-mediated demyelinating neuropathies was used to demonstrate that SM identifies myelin breakdown in these patients.

## Results

Our major objective was to identify a fast, reliable and feasible myelin biomarker. Therefore, we had to: i) identify a marker sensitive and specific to detect even small myelin rearrangements using an assay of widespread application; ii) demonstrate the value of this myelin biomarker for human patients.

### SM monitors peripheral myelination during development and small myelin rearrangements

**Figure 1:**
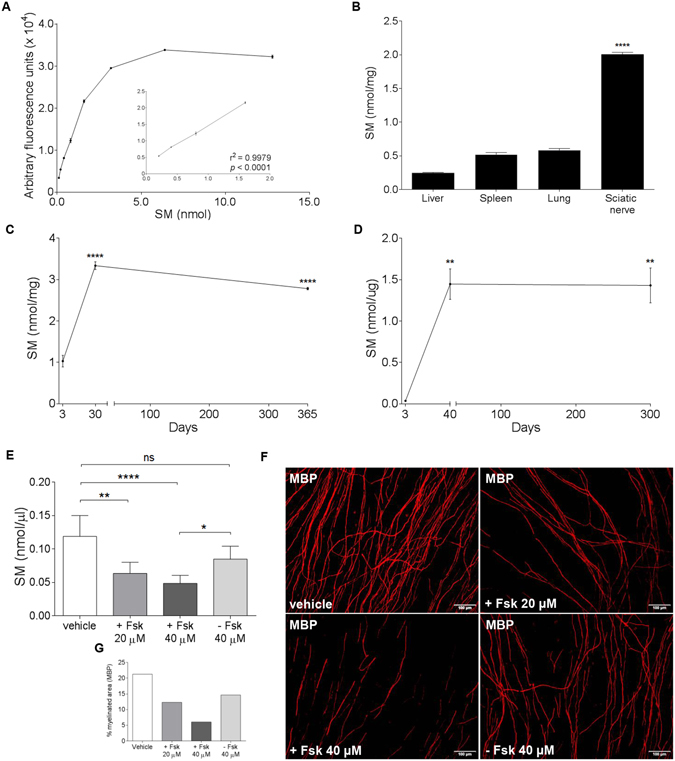
SM levels mirror both peripheral myelination during development and small myelin rearrangements (**A**) SM titration curve of a fluorescence-based assay showed a significant and reliable linearity from 0.2 to 1.6 nmol. (**B**) Quantification of SM in several tissues from 270-day-old rats displayed a specific enrichment in sciatic nerves. (**C**,**D**) SM levels in rat sciatic nerves and purified myelin progressively increased during development (sciatic nerve: 3-day-old, n = 5; 30-day-old, n = 8; 365-day-old, n = 3; purified myelin: 3-day-old, n = 3; 40-day-old, n = 3; 300-day-old, n = 3). (**E**). We treated DRG cultures with Forskolin (Fsk) 20 and 40 μM to induce demyelination and then let them recover by Fsk removal. We found that SM was able to detect both demyelination and remyelination (n = 6). (**F**,**G**) Immunofluorescence staining of myelin by MBP in DRG cultures and quantification of MBP-positive myelinated fibers supported biochemical data. Scale bars: 100 µm. Errors bars in A, B represent the SD; *p < 0.05, **p < 0.01, ***p < 0.001, ****p < 0.0001, ns = not-significant. Goodness of fit (r^2^) was determined by linear regression analysis. Holm-Sidak multiple comparison test after 1-way analysis of variance was used for statistical comparison in B-E.

We optimised, in peripheral myelinated tissues and cultures, a fluorescence-based assay to quantify SM, an essential structural and functional component of the myelin membranes^9^. First, we established a standard curve for SM to obtain absolute concentration (nmol) from fluorescence signal. This curve showed both a linear regression from 0.2 to 1.6 nmol of input SM and high sensitivity at very low concentrations (<0.02 nmol) (Fig. 1A and Supplementary Figure S2). At concentrations higher than 1.6 nmol the curve was less linear and fits a hyperbola regression equation understimating the SM amount.

To demonstrate SM specific enrichment in the myelin sheath, we compared its levels in a myelinated tissue of PNS, namely sciatic nerve, with those of other non-myelinated tissues (liver, spleen and lung) of adult rats. As shown in Fig. 1B, sciatic nerves contained five times more SM than non-myelinated tissues. Moreover, we quantified SM during postnatal development in sciatic nerves at different time points (i.e. 3-, 30-, 365- day-old rats). We found a massive enrichment of SM content during the first 30 days after birth, mimicking the timing of internode maturation of the myelin sheath (Fig. 1C). Afterwards, as already known for myelin, SM stabilised up to one year^10^. To further address this point, we also measured cholesterol levels in the same nerves^11^. Interestingly, even cholesterol, that is rate-limiting for the development of the myelin sheath, showed the same trend over time (Supplementary Figure S1). In addition, to further strengthen the specificity of SM to monitor the myelin membrane, we quantified its levels in purified myelin extracts from rat sciatic nerves at similar time points (i.e. 3-, 40-, 300-day-old rats). Again, we found a remarkable increase of SM up to 40 days (Fig. 1D).

Finally, to demonstrate that SM was able to monitor even small myelin rearrangements, we quantified its levels in a chemically induced *in vitro* model of peripheral demyelination and remyelination^12^. The decrease of SM content closely paralleled dose-dependent demyelination elicited by forskolin (Fsk) supplementation to myelinating dorsal root ganglia (DRG) cultures. Notably, remyelination occurring after Fsk removal, was matched with a corresponding increase of SM levels (Fig. 1E–G).

### SM is a biomarker of myelin breakdown in human CSF

**Figure 2:**
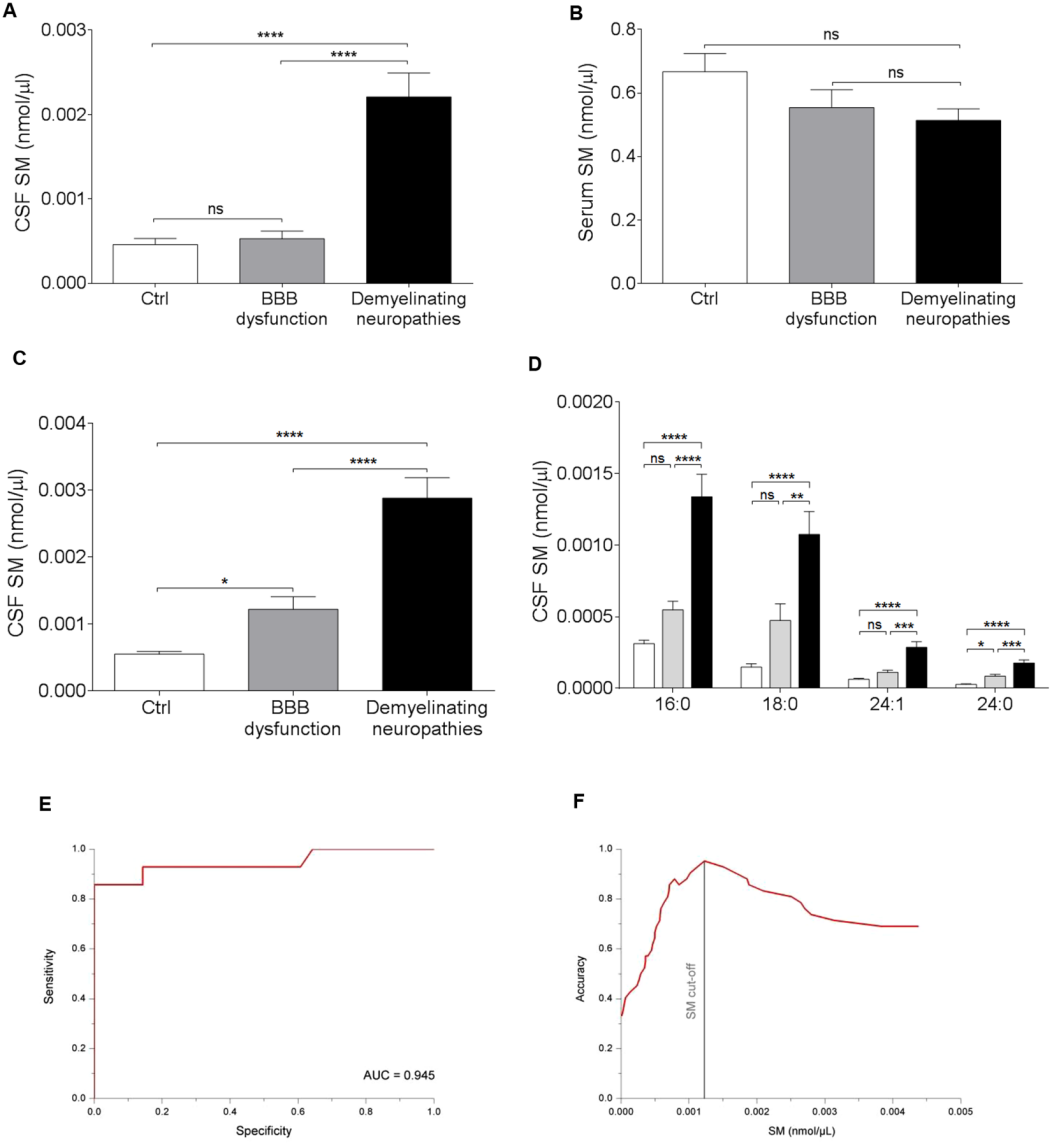
SM is increased in patients affected by demyelinating neuropathies. (**A**,**B**) We assessed SM in clinically well-characterized subgroups of patients affected by: definite demyelinating neuropathies, namely GBS and CIDP (Black bar, Demyelinating neuropathies, n = 14), neurological disorders with blood brain barrier dysfunction but not overt demyelination (Grey bar, BBB dysfunction, n = 13), and other non-demyelinating neurological disorders (White bar, Ctrl, n = 15). We demonstrated that SM was significantly increased in patients with demyelinating neuropathies compared to both the control groups. Interestingly, SM exclusively increased in the CSF of these patients to exclude a direct exchange with peripheral blood. (**C**,**D**) LC-MS/MS targeted-sphingomyelin analysis showed that the total SM species contribution is definitely consistent with those obtained by the fluorescence-based assay. 16:0, 18:0, 24:0, 24:1 are the most abundant SM species with different saturated and unsaturated fatty acid chains. (**E**) Specificity and sensitivity of SM assay were tested by ROC curve analysis (**F**) A cut-off value for SM was also calculated. ***p < 0.001, ****p < 0.0001, ns = not-significant. Holm-Sidak multiple comparison test after 1-way analysis of variance was used for statistical comparison in (**A**–**D**). ROC implementation in Mathworks MATLAB environment was used in **E, F**.

After obtaining evidence that SM is a myelin marker in experimental models, we tested our hypothesis on the CSF of patients affected by immune-mediated demyelinating neuropathies. In particular, we speculated that demyelination at tissue level might be detected by SM dosage in the CSF of these patients^13–15^. In fact, proximal nerve roots located in the subarachnoid region are in close contact with CSF. Therefore, the altered composition of CSF could mirror the damage within the tissue of the nervous system^16^. First, we optimised a fluorescence-based assay to quantify SM in CSF that we routinely collect for clinical practice (Supplementary Figures S2 and S3). Then, we quantified SM in a cohort of patients with definite immune-mediated, demyelinating neuropathies in which myelin breakdown occurs also in the nerve roots in close contact with CSF. To exclude a role of blood brain barrier (BBB) in the determination of CSF SM levels, patients affected by other non-demyelinating neurological disorders and patients showing obvious BBB dysfunction but not overt demyelination were used as two distinct control groups. Interestingly, we found that SM was significantly higher in the CSF of patients affected by demyelinating neuropathies both compared to the control (p = 3.81 * 10 − 8) and BBB dysfunction (p = 1.34 * 10 − 7) groups (Fig. 2A). Most importantly, in all patients the contribution of serum-deriving SM was not relevant, excluding a passive diffusion from the periphery (Fig. 2B).

To assess the specificity of the fluorescence-based assay, we analysed the same CSF samples by liquid chromatography tandem mass spectrometry (LC-MS/MS), an advanced technique able to quantify any lipid metabolites. Interestingly, a detailed targeted-SM analysis confirmed the striking increase of the most represented SM species in the CSF of patients with peripheral demyelination (Fig. 2C,D).

To test the discriminatory power of SM to identify demyelination in CSF, we ran a receiver operating characteristic (ROC) curve analysis and defined the optimal cut-off value in the previously defined groups of patients. We found that the area under the curve (AUC) for SM was 0.945, indicative of a good discriminatory biomarker. The SM cut-off for optimum sensitivity and specificity was 0.00118 nmol/µl (Fig. 2E,F).

Moreover, we correlated SM with available neurophysiology in 12 out of 15 patients affected by peripheral demyelinating neuropathies. A significant inverse correlation was found between SM and peroneal motor conduction velocity (MCV). Instead, SM did not correlate with amplitude of compound motor action potential (cMAP) of the same nerves (Supplementary Figure S4).

**Figure 3:**
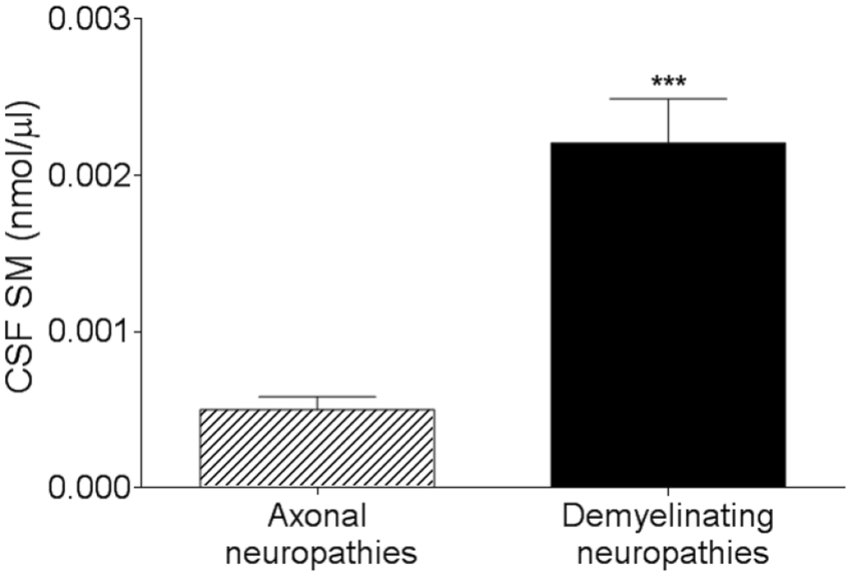
SM is able to distinguish axonal from demyelinating neuropathies. SM alone was significantly increased in patients affected by demyelinating neuropathies (n = 14) compared to those affected by axonal neuropathies (n = 8). ***p < 0.001. Unpaired 2-tailed t-test was used for statistical comparison.

Notably, SM alone was able to distinguish demyelinating from axonal neuropathy. In fact, when we compared patients with peripheral demyelination both acute (Guillain-Barrè syndrome, GBS) and chronic (chronic inflammatory demyelinating polyradiculoneuropathy, CIDP) with those showing axonopathy, we found a significant difference between the two groups (Fig. 3).

**Figure 4:**
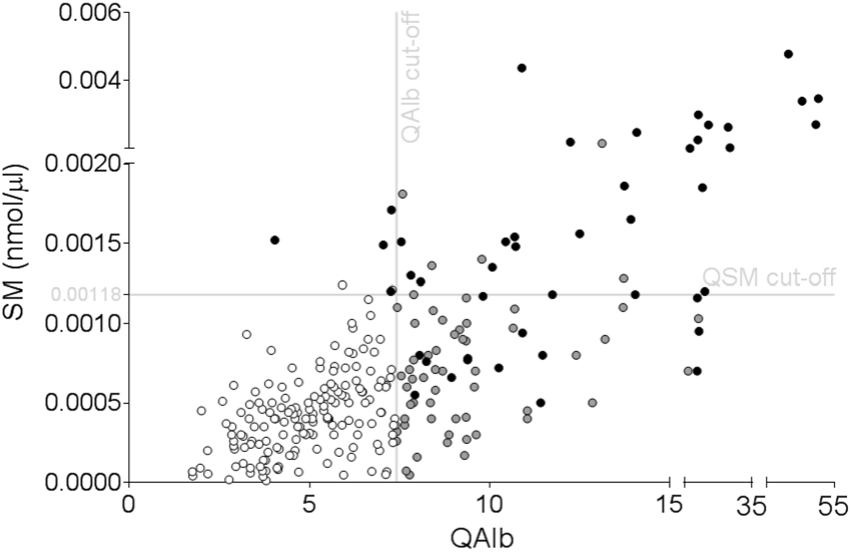
SM is a biomarker of myelin breakdown in the CSF of human patients. A scatter plot of all the 262 analysed patients based on QAlb and SM cut-off (X and Y axis, respectively) is shown. Notably, most of the patients with peripheral demyelination (Black circles) were over the SM threshold. Control subjects, either with or without BBB dysfunction (Grey and white circles, respectively) were also reported in the graph. Grey lines represent SM (Y axis) and QAlb (X axis) cut-off.

Next, we generated a graph in which we plotted for each of the 262 analysed samples SM and albumin quotient (QAlb) values, resembling Reiber’s diagram^17^. Patients were not randomly distributed but clustered in at least three groups (Fig. 4). Based on discharge report, among patients with SM over the cut-off, 31 out of 39 patients showed clinical and neurophysiological evidence of peripheral demyelination. Accordingly, among patients with SM under the cut-off, only 15 out of 223 patients displayed sign of peripheral demyelination, independently from the presence or absence of BBB dysfunction (Table 1).

**Table 1:** Patients distribution according to SM values.

Patient CSF data	↓QAlb	↑QAlb	
↓SM	158 (1/158 Demyelinated)	65 (14/65 Demyelinated)	223 (15/223 Demyelinated) **7%**
↑SM	6 (4/6 Demyelinated)	33 (27/33 Demyelinated)	39 (31/39 Demyelinated) **79%**
			262 Total patients

↓SM = CSF Sphingomyelin under the cut-off; ↑SM = CSF Sphingomyelin over the cut-off; ↓QAlb = CSF QAlb index under the cut-off; ↑QAlb = CSF QAlb index over the cut-off.

To support the potential novelty of this marker, we also compared SM with QAlb and immunoglobulins G quotient (QIgG) in patients showing BBB dysfunction but not demyelination. We focused on this group as the peripheral demyelinating patients were positive either for QAlb and SM, thus making awkward separate demyelination from inflammation. SM did not correlate with QIgG and poorly with QAlb in 57 patients. Conversely, as expected, QAlb and QIgG strongly correlated in the same patients (Supplementary Figure S5).

## Discussion

A growing number of studies proved several biomarkers to be associated with the pathogenesis, development, and even recovery of demyelinating neuropathies, although critical issues emerge which restrict their widespread acceptance in clinical practice^16^.

In this study, we have explored a lipid myelin biomarker by a simple, inexpensive, reliable, and rapid fluorescence-based assay. In fact, a limiting factor for myelin disorders is the lack of de-remyelination biomarkers specific and sensitive to monitor myelin remodeling, to test putative pro-remyelinating compounds and thereby select specific treatments. Moreover, myelin is mainly composed by lipids and their derangement has been demonstrated in most of these diseases^9, 18–20^. In particular, we focused on SM due to specific enrichment and essential structural role in myelin sheath, both in CNS and PNS^9, 21^.

Our first interest was to demonstrate that SM levels monitor the myelin amount. Our results clearly show that SM assay is able to detect and quantify myelin content in normal rodent peripheral nerves and purified myelin during development, and subtle changes of myelin levels in an *in vitro* model of peripheral demyelination-remyelination. This finding is extremely relevant in different conditions including pre-clinical trials in which may be awkward to monitor small myelin rearrangements following pharmacological and non-pharmacological therapies. Indeed, to definitely demonstrate that SM dosage is able to monitor remyelination *in vivo*, namely in the CSF, additional experiments are needed on animal models in which peripheral de- and remyelination can be exactly and reliably monitored.

To realistically translate these results to humans, we had to test this myelin biomarker in biological fluids. In neurological patients, peripheral blood and CSF are the main and ideal source of biomarkers^8, 22^. In fact, nervous tissue exchanges metabolites with blood and CSF; demyelinating processes, causing the breakdown of the myelin membranes to individual constituents, is associated with the liberation of them to the blood/CSF compartments^13–15^. Thus, we hypothesized that demyelination at tissue level might be detected by SM dosage in the CSF. Therefore, to test our hypothesis, we selected patients affected by immune-mediated demyelinating polyradiculoneuropathies in which myelin breakdown may involve spinal roots and is therefore in close contact to CSF; more importantly, spinal tap is routinely performed for diagnostic purposes in these cases^16^. We found that SM is significantly increased in the CSF of these patients compared to all the control groups (Fig. 2A). Our result is even more interesting as SM does not change in the serum, excluding the contribution of peripheral SM (Fig. 2B). Moreover, SM-targeted analysis by LC-MS/MS confirmed the increased levels of SM species in the CSF of patients with peripheral demyelination. Therefore, we can conclude that the performance of our low-cost assay is in line with much more expensive techniques, in terms of accuracy and sensitivity in quantification of the total SM content.

As retrospective, cross-sectional studies, like this one, have significant limits, we refined our approach to sustain the conclusions. First, the accuracy of our results was quantified by ROC curve analysis that demonstrated a good discriminatory power for SM and allowed to define a SM cut-off.

Then, based on these results, we extended the analysis up to 262 CSF samples collected from neurological patients. Notably, 79% of patients with SM over the threshold show signs of peripheral demyelination, while only 7% of those with low SM present peripheral myelin deficit (Table 1). Interestingly, patients with higher SM represented 15% of the entire population, identifying a novel, substantial and previously undistinguishable group.

Inside the whole population, 31 patients were affected by axonal neuropathy of different etiology, including diabetic and chemotherapy-induced neuropathies. Interestingly, 28 of them (90%) showed SM under the threshold. Thus, SM alone distinguishes demyelinating neuropathies from other non-demyelinating neurological disorders, including axonal neuropathies. To the best of our knowledge, these two clinical phenotypes are currently identify only by neurophysiology but simple and reliable methods are not available. In our opinion, this is the first of possible applications of SM assay in clinical practice.

Concerning patients in which SM levels do not match with the clinical phenotype, we speculate that one reason may lie in the restricted sample size used to define SM cut-off. In fact, several of these patients are located close to SM threshold and they would be likely correctly recognized by a more realistic cut-off which can be only calculated on a wider population of well-characterized demyelinating patients. Finally, at present it is quite difficult to compare SM with quantitative markers of myelin damage as there are no gold standards or validate biomarkers in this field.

We also wondered whether SM overlapped with QAlb and QIgG, parameters usually associated to BBB dysfunction and inflammation^17^. Indeed, we demonstrated that SM discerns an original group of patients independently from commonly used CSF indexes (Supplementary Figure S5). This is a relevant issue because most of demyelinating disorders have a strong inflammatory/immunological component and a specific and reliable myelin biomarker, able to separate demyelination from inflammation, is crucial and highly needed.

Another critical issue is that clinical application of many biomarkers may be limited by expensive methods, invasive examinations and low sensitivity/specificity^16^. The SM assay largely satisfies these requirements. In fact, it is: i) simple and easily amenable to routine utilization without the need for sophisticated equipment or operator skill; ii) fast, allowing the simultaneously evaluation of more than one hundred samples; iii) inexpensive, with a total estimated cost of five euro/sample; iv) reliable, being measured with acceptable accuracy, precision, robustness, and reproducibility, as shown in this study.

To definitely assess the clinical relevance of SM, a multicenter, prospective longitudinal study would be advisable to capture a larger number of patients affected by immune-mediated demyelinating neuropathies. To further strengthen the meaning and value of this myelin biomarker, it will be extremely interesting to translate these evidences to central demyelination, namely multiple sclerosis. This would allow us to candidate SM assay as a novel CSF index to be routinely used in neurological clinical practice.

## Methods

### Animals

Sprague-Dawley rats were used for the experiments. Research protocols presented in this study are conducted in accordance with the ARRIVE guidelines, and are included in those reviewed and approved by the OPBA (Institutional Animal Welfare Body) and by the Italian Ministry of Health.

### Myelin purification

A light-weight membrane fraction enriched for myelin was purified from rat sciatic nerves homogenized in 0.27 M sucrose according to Larocca and Norton^23^.

### *In vitro* peripheral demyelination/remyelination

A demyelination/remyelination experimental setting established on myelinating DRG cultures was used. Briefly, Fsk supplementation of myelinating DRG cultures causes demyelination that is reversed by Fsk withdrawal^12^. DRG cultures were used either for immunofluorescence staining of the myelin sheath and SM dosage^24, 25^. In particular, myelin staining was performed using a primary monoclonal antibody against the myelin basic protein (MBP) (MAB 382, Merck Millipore) and a secondary goat anti-mouse ALEXA594-IgG antibody, as previously described^24^. Images were taken with an Olympus PROVIS AX60 microscope, connected to an Olympus DP70 digital camera. To quantify MBP, we calculated the percentage of myelinated area expressed as the area of pixels positive for the red staining of MBP compared to the total area of the acquired image. To quantify SM, cultures were scraped and recovered from the culture dishes by centrifugation. Resulting pellets were hand-held homogenized in 0.25% Triton X-100 and stored at −80 °C until lipid extraction and SM dosage.

### Subjects

We performed a cross-sectional retrospective study on the CSF of 262 neurological patients collected between 2014 and 2015 from our laboratory of analysis. The Regional Ethics Committee IRCCS AOU San Martino – IST (CER Liguria) approved the current study protocol. Informed consent to use CSF and serum was obtained from all the patients involved in the study. We examined CSF of patients authorizing access to personal health information according to the Helsinki Declaration as revised in 2013. Samples of patients positive for HCV, HIV and bacterial and viral encephalitis and meningitis were excluded from the study. Samples of patients affected by suspected or definite multiple sclerosis were not included in the present study, as they will be discussed in a dedicated paper. We used three well-characterized sub-groups of patients to define the sensitivity and specificity of SM assay and the cut-off value. The first sub-group was represented by typical inflammatory demyelinating neuropathies, namely GBS (AIDP variant) and CIDP (n = 14). The diagnosis of definite GBS was based on published criteria, whereas CIDP diagnosis was performed according to the criteria of the European Federation of Neurological Societies/Peripheral Nerve Society^26, 27^. We used, as control subjects, 15 patients affected by other non-demyelinating disorders including hydrocephalus (n = 1), myalgia (n = 2), dizziness (n = 1), motor neuron disease (n = 2), epilepsy (n = 2), cognitive impairment (n = 3), meningioma (n = 1), chronic vascular encephalopathy (n = 1) and axonal neuropathy (n = 2). Finally, 13 patients with obvious BBB dysfunction, as shown by CSF analysis and affected by non-demyelinating diseases, according to instrumental and clinical data, were used as a second group of control. These patients included, motor neuron disease (n = 1), cognitive impairment (n = 3), chronic vascular encephalopathy (n = 3) and axonal neuropathy (n = 6).

MCV and cMAP, recorded on peroneal nerves at the time of spinal tap, were considered and related to SM levels only for the sub-group of demyelinating neuropathies as neurophysiological studies are part of diagnostic criteria for these patients.

### Human sample treatment and storage

CSF (3 ml) and peripheral blood (5 ml) were collected from all the subjects involved in the study and immediately sent to the laboratory for routinely diagnostic purposes. Immunochemical examination of CSF and serum was performed according to the recommendations of the Italian Association for Neuroimmunology^28^. In particular, the CSF to serum albumin concentration quotient (QAlb) is routinely used to evaluate blood-CSF barrier integrity. QAlb values higher than the cut-off suggested by international guidelines are considered positive for BBB dysfunction. The CSF to serum immunoglobulin G concentration quotient (QIgG) reflects both the rate of IgG permeability into the CSF from the blood and the intrathecal synthesis of IgG^29^. Samples were stored at RT during these steps. Exceeding amounts of CSF (at least 1 ml of CSF and 1 ml of peripheral blood serum) were sent to the Neuropathology Laboratory of DINOGMI the same day of spinal tap, and immediately frozen and stored at −80 °C in 500 μl aliquots to perform SM dosage, avoiding repeated freezing and thawing. This latter procedure is fundamental to preserve lipid and protein integrity over time and consistent with the guidelines for uniform reporting of body fluid biomarker studies in neurologic disorders^30^.

### Lipid extraction

Lipids were extracted from rat sciatic nerve, liver, spleen, and lung and purified myelin, following the detergent/heating method^25^. DRG homogenates and human CSF lipids were extracted following the Bligh and Dyer method^31^.

### SM fluorescence-based assay

We recently optimised a fluorescence-based assay to quantify SM in tissue homogenates and biological fluids. All chemicals used in the following procedures were purchased by Sigma-Aldrich. This assay is based on lipid extraction from tissues, cells and fluids followed by enzymatic reactions consisting in the following steps: 1) hydrolysis of SM to phosphorylcholine and ceramide by sphingomyelinase; 2) hydrolysis of phosphorylcholine by alkaline phosphatase yielding choline and 3) oxidation of choline by choline oxidase with formation of hydrogen peroxide and betaine. Hydrogen peroxide, in the presence of horseradish peroxidase is able to react 1:1 with dihydroxyphenoxazine (AR) to generate resorufin, a highly fluorescent product^25, 32^. In particular, 5–10 μl of lipid extract from rat tissue homogenates and DRG cultures, human CSF and serum was added to individual wells of a 96-well microtiter plate that contained an enzymatic cocktail consisting of 12.5 mU of *Bacillus cereus* sphingomyelinase, 400 mU of alkaline phosphatase, 120 mU of choline oxidase, 200 mU of horseradish peroxidase, and 20 nmol of AR in 100 μl of reaction buffer (50 mM Tris-HCl, 5 mM MgCl2, pH 7.4). For each sample, the relative negative control obtained removing sphingomyelinase by the reaction mixture was also analyzed. After 20 min incubation at 37 °C in the dark, the microtiter plate was read using a fluorescence microplate reader with excitation and emission wavelength at 560 and 587 nm, respectively (Infinite 200 PRO, Tecan Italia Srl). A standard curve was prepared by making serial dilutions (from 0.0125 to 1.6 nmol) from a 2.8 nmol/μl of SM standard stock solution. For each sample SM levels were calculated from the difference in fluorescence between the sample and its relative negative control. Resulting values were interpolated with the related standard curve to obtain absolute SM concentration (nmol). SM levels for tissues homogenates, purified myelin, and DRG cultures were normalized to the weight, total protein content, and total volume, respectively.

### Quantification of sphingomyelins by LC-MS/MS

Targeted analysis of sphingomyelins was performed using the method already published^33^.

### Statistical analysis

Results are presented as mean ± SEM unless otherwise specified. Goodness of fit (r^2^) was determined by linear regression analysis. Statistical differences between two groups were determined using the two-tailed Student’s t-test. Multiple group comparison was performed by one-way ANOVA followed by the Holm-Sidak test. Correlation coefficient was estimated by the Spearman’s rank correlation test. We evaluated our test performances in terms of accuracy by using the ROC curve. In particular, we used our customized ROC implementation in Mathworks MATLAB environment^34^. Statistical differences were considered to be significant when p < 0.05. All statistical analysis was performed using the Graph Pad 7.0 (Prism) software.

## Electronic supplementary material

Supplementary info
