# Supplementary material for: Sphingomyelin as a myelin biomarker in CSF of acquired demyelinating neuropathies
Source: Sci Rep. 2017 Aug 10;7:7831. doi: 10.1038/s41598-017-08314-1 (PMC5552737; doi:10.1038/s41598-017-08314-1)
Supplement: Supplementary file 1 — Supplementary info [file 41598_2017_8314_MOESM1_ESM.pdf]

Sphingomyelin as a myelin biomarker in CSF of acquired demyelinating neuropathies

Giovanna Capodivento, Davide Visigalli, Martina Garnero, Roberto Fancellu, Michela Demetra Ferrara, Abdul Basit, Zeeshan Hamid, Vito Paolo Pastore, Silvano Garibaldi, Andrea Armirotti, Gianluigi Mancardi, Carlo Serrati, Elisabetta Capello, Angelo Schenone, and Lucilla Nobbio

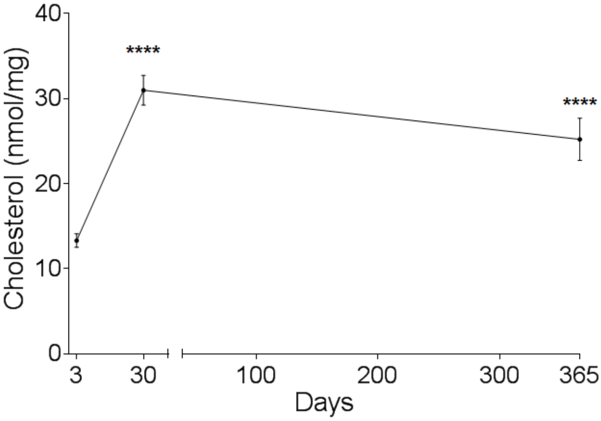

Supplementary Figure S1: SM during development resembles cholesterol levels, another essential myelin component. We monitored over time cholesterol in the same sciatic nerves used for SM dosage by a specific fluorescence-based assay (Amundson & Zhou, 1999). Cholesterol levels in rat sciatic nerves progressively increased during development (sciatic nerve: 3-day-old, n = 5; 30-day-old, n = 8; 365-day-old, n = 3). ****$p < 0.0001$. Holm-Sidak multiple comparison test after 1-way analysis of variance was used for statistical comparison.

A

Exogenous SM (d18:1/17:0), 1µM final concentration, N=5		
Reference	CSF	Percent Recovery
1,09±0,07 µM	1,07±0,12 µM	82-117 %

B

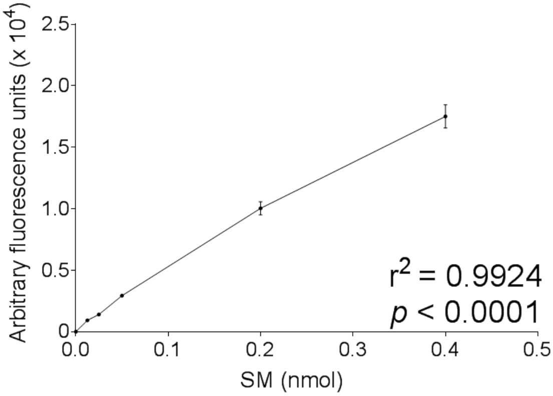

Supplementary Figure S2: Recovery of SM extraction from human CSF and standard curve. (A) We used the exogenous d18:1/17:0 SM, spiked into human CSF to a final 1µM concentration. The table reports the average measured concentration of this lipid in neat solvent (9:1 CH3OH:CHCl3) and in human CSF, in quintuplicate. Quantification was performed using a standard calibration curve. The observed recovery of the exogenous standard from human CSF is in line with already published papers. (B) (A) SM titration curve showed a significant and reliable linearity for low concentrations from 0.0125 to 0.4 nmol.

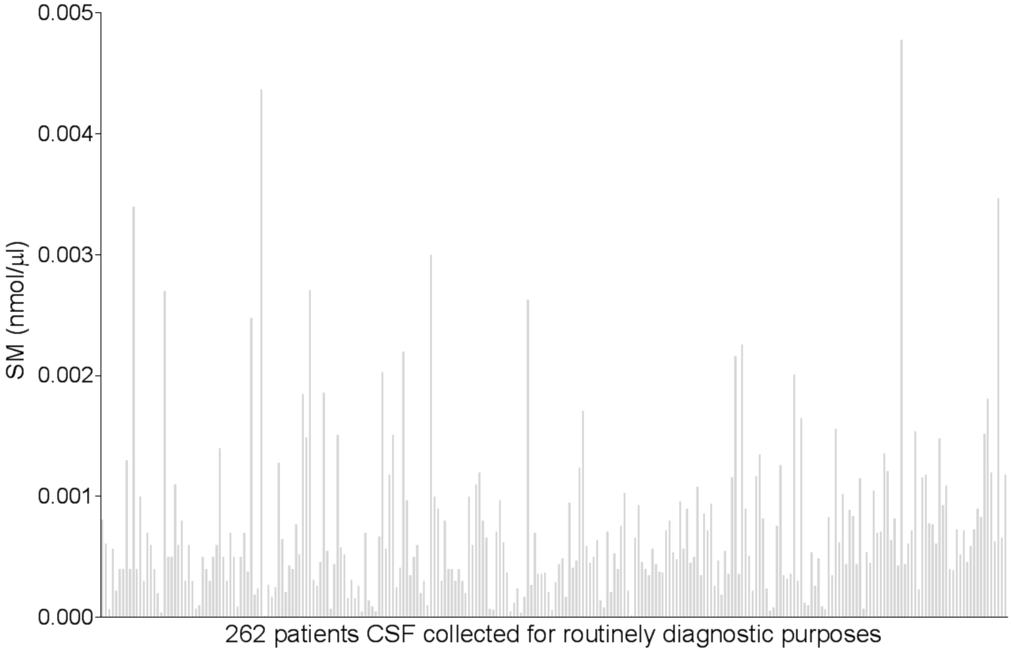

Supplementary Figure S3: SM is quantiafiable in the CSF of human patients by a fluorescence-based assay. We optimised a fluorescence-based assay to rapidly and reliably quantify SM in the CSF of human patients. SM levels were extremely variable to suggest a potential clinical relevance for patients affected by neurological disorders.

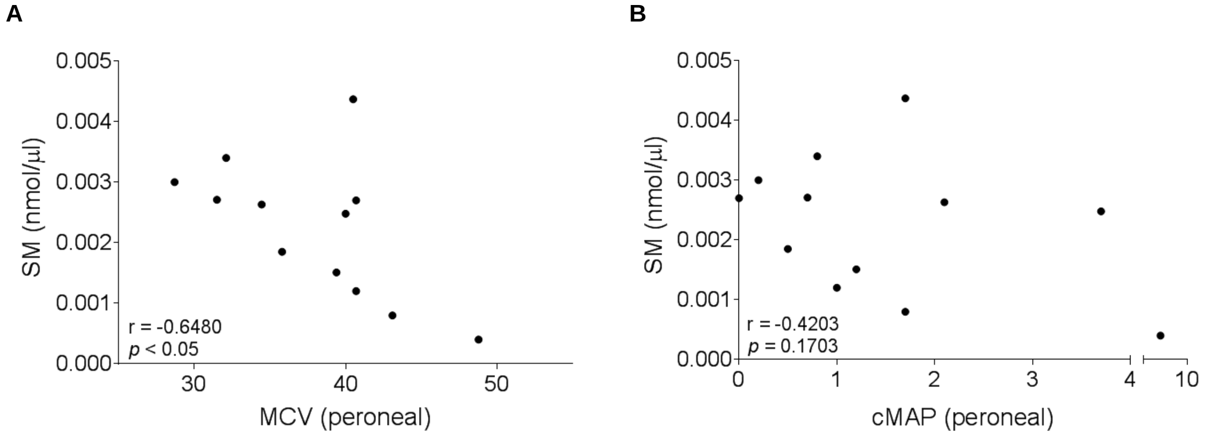

Supplementary Figure S4: SM inversely correlated with motor conduction velocity in demyelinating patients. We correlated SM with motor conduction velocity (MCV) and amplitude of compound motor action potential (cMAP) of the peronel nerve in patients affected by demyelinating neuropathies (n = 12). (A) We observed an inverse correlation between SM and peroneal MCV to support SM ability to detect myelin rearrangements. (B) No correlation was observed between SM and peroneal cMAP. Spearman's rank correlation test was used for statistical analysis.

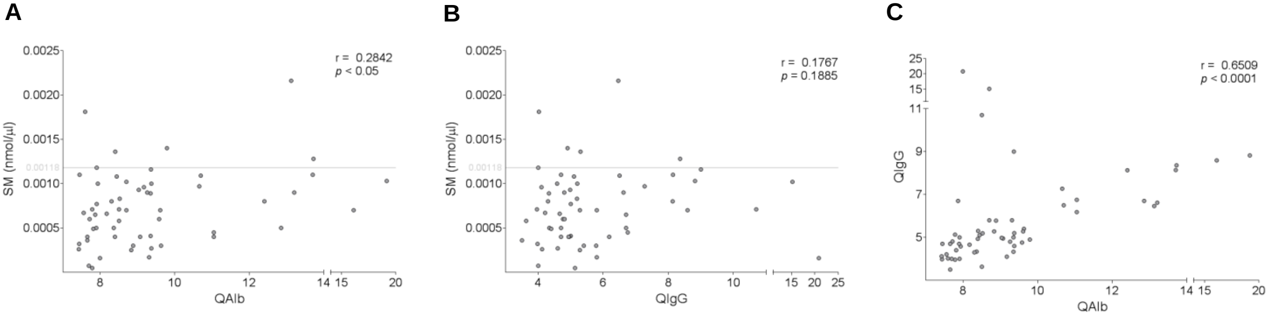

Supplementary Figure S5: SM discerns an original group of patients independently from commonly used CSF indexes. To better appreciate the novelty of this index, we correlated SM with QAlb and QIgG in patients showing BBB dysfunction but not demyelination (n = 57). In fact, in these patients we were able to eventually identify a direct relationship between SM and currently used CSF laboratory indexes without undesired overlapping. (A, B) We did not find any correlation between SM and QIgG and a poor correlation between SM and QAlb. (C) As expected, in the same patients, a strongly significant correlation was found between BBB dysfunction (QAlb) and synthesis of immunoglobulins G (QIgG). Spearman's rank correlation test in A-C was used for statistical analysis.
